# Proposal of a Clinical Decision Tree Algorithm Using Factors Associated with Severe Dengue Infection

**DOI:** 10.1371/journal.pone.0161696

**Published:** 2016-08-23

**Authors:** Jayashamani Tamibmaniam, Narwani Hussin, Wee Kooi Cheah, Kee Sing Ng, Prema Muninathan

**Affiliations:** 1 Department of Medicine, Taiping Hospital, Perak, Malaysia; 2 Clinical Research Centre, Taiping Hospital, Perak, Malaysia; 3 Department of Medicine, Kuala Lumpur Hospital, Kuala Lumpur, Malaysia; University of Malaya, MALAYSIA

## Abstract

**Background:**

WHO’s new classification in 2009: dengue with or without warning signs and severe dengue, has necessitated large numbers of admissions to hospitals of dengue patients which in turn has been imposing a huge economical and physical burden on many hospitals around the globe, particularly South East Asia and Malaysia where the disease has seen a rapid surge in numbers in recent years. Lack of a simple tool to differentiate mild from life threatening infection has led to unnecessary hospitalization of dengue patients.

**Methods:**

We conducted a single-centre, retrospective study involving serologically confirmed dengue fever patients, admitted in a single ward, in Hospital Kuala Lumpur, Malaysia. Data was collected for 4 months from February to May 2014. Socio demography, co-morbidity, days of illness before admission, symptoms, warning signs, vital signs and laboratory result were all recorded. Descriptive statistics was tabulated and simple and multiple logistic regression analysis was done to determine significant risk factors associated with severe dengue.

**Results:**

657 patients with confirmed dengue were analysed, of which 59 (9.0%) had severe dengue. Overall, the commonest warning sign were vomiting (36.1%) and abdominal pain (32.1%). Previous co-morbid, vomiting, diarrhoea, pleural effusion, low systolic blood pressure, high haematocrit, low albumin and high urea were found as significant risk factors for severe dengue using simple logistic regression. However the significant risk factors for severe dengue with multiple logistic regressions were only vomiting, pleural effusion, and low systolic blood pressure. Using those 3 risk factors, we plotted an algorithm for predicting severe dengue. When compared to the classification of severe dengue based on the WHO criteria, the decision tree algorithm had a sensitivity of 0.81, specificity of 0.54, positive predictive value of 0.16 and negative predictive of 0.96.

**Conclusion:**

The decision tree algorithm proposed in this study showed high sensitivity and NPV in predicting patients with severe dengue that may warrant admission. This tool upon further validation study can be used to help clinicians decide on further managing a patient upon first encounter. It also will have a substantial impact on health resources as low risk patients can be managed as outpatients hence reserving the scarce hospital beds and medical resources for other patients in need.

## Introduction

Dengue is the most common mosquito-borne viral disease of international public health concern[1]. The clinical manifestation ranges from sub-clinical to the more life threatening dengue hemorrhagic fever (DHF) and dengue shock syndrome (DSS)[2]. To improve on disease severity identification, WHO proposed a new classification in 2009: dengue with or without warning signs and severe dengue[2].

In Malaysia, dengue has been imposing a huge economical and physical burden on the local hospital settings. From a total of 7103 reported cases in the year 2 000, the number has increased to 108 698 in the year 2014, a 15 fold rise[3]. The total number patients admitted for dengue fever in government hospitals in Malaysia in the year 2013 was 42 701, whereas the very next year, the number rose to a staggering 101 131[4]. In the US, it is estimated to cost a mean of USD 317+/- 105 for an ambulatory case and USD 947+/- 389 for hospitalized case[5]

Lack of a simple tool to differentiate mild from life threatening infection may have led to unnecessary hospitalization of dengue patients. In a single-center outpatient based prospective study, only 30.7% of the admitted dengue cases developed DHF [6].

Therefore, we aim to look at the laboratory and clinical determinants in our cohort of patients that is associated to severe dengue. We then proposed a simple algorithm tree using the significant determinants which may be used by physicians to aid in the decision on admissions of patients presenting with dengue fever.

## Methodology

We have registered this study with the National Medical Research Register (NMRR) and obtained an ethical approval from Malaysian Research Ethical Committee (MREC) with the reference number NMRR-15-1116-26733. This was a single-centre, retrospective study involving serologically confirmed dengue fever patients, admitted to Ward 20 (B) of Hospital Kuala Lumpur from 1st February to 31st May 2014 (4 months duration). No consent was obtained from patients to maintain patient’s confidentiality. All patients’ information was anonymized and de-identified prior to analysis.

Definition of severe dengue is based on WHO 2009 criteria [2]. Patient is considered as severe dengue when he/she presents with dengue with at least one of the following criteria:

Severe plasma leakage leading to:

- Shock (DSS)
- Fluid accumulation with respiratory distress
- Severe bleeding as evaluated by clinician
- Severe organ involvement
- Liver: AST or ALT ≥ 1000
- CNS: impaired consciousness
- Failure of heart and other organs

Data was collected using a data collection sheet. Variables include socio demography, co-morbidity, days of illness before admission, symptoms, warning signs, vital signs and laboratory result (full blood count, liver function test, blood urea and serum creatinine). The outcomes were severe dengue and death. Demographic and clinical data were obtained on admission, while dengue severity was obtained at discharge.

Descriptive statistics was tabulated using frequency and mean with standard deviation. Simple and multiple logistic regression analysis were done to determine significant risk factors associated with severe dengue. Based on those factors, decision tree algorithm for severe dengue was plotted. Analysis was done using SPSS version 21.

## Results

### Demographic and clinical profiles of dengue patients

**Fig 1:**
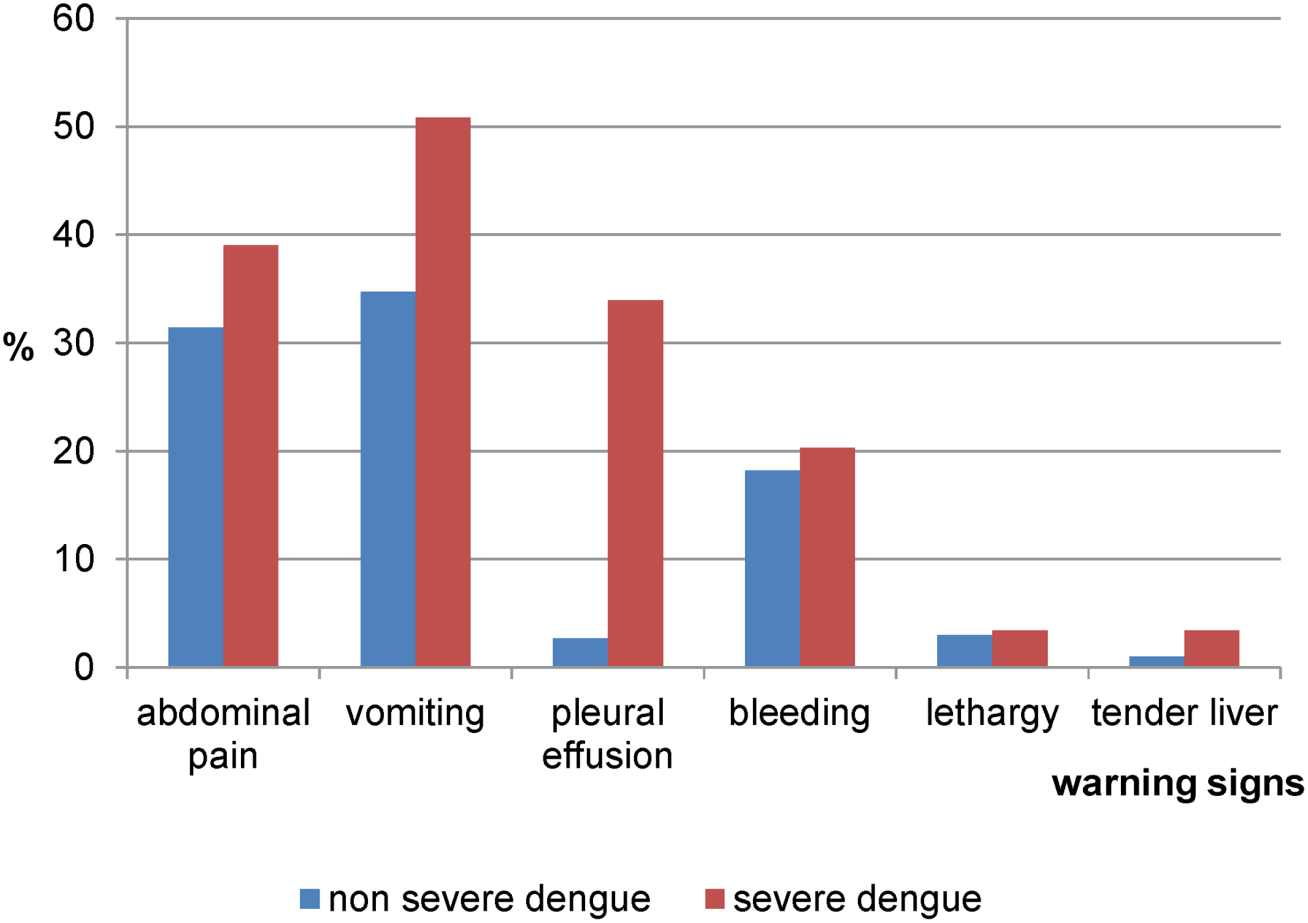
Percentage of warning signs by dengue severity.

This study included 657 patients. The demographic profiles are shown in Table 1. The mean duration of illness before admission was 5 (1.63) days. 22.5% had at least one co-morbid with hypertension as the commonest disease. Overall, the commonest warning signs were vomiting (36.1%) and abdominal pain (32.1%). Of the 657 patients, 59 (9.0%) had severe dengue (Fig 1). All patients were discharged alive.

**Table 1:** Demographic profiles of dengue patients.

Variables	Frequency	%
Age		
Less or equal 60 years	614	93.5
More than 60 years	43	6.5
Ethnicity		
Malay	432	65.8
Chinese	61	9.3
Indian	88	13.4
Others	76	11.5

On admission, both groups were significantly different in their systolic blood pressure and few lab results (Table 2). For severe dengue group, more patients had low systolic blood pressure than the non-severe dengue group. They also had more patients with higher haematocrit, low albumin and higher urea.

**Table 2:** Vital signs and laboratory results based on dengue severity.

Variables	Frequency (%)		p value*
	Non severe dengue	Severe dengue	
**Systolic BP**			
Less than 90 mmHg	9 (2.2)	8 (18.2)	<0.001
More or equal 90 mmHg	402 (97.8)	36 (81.8)	
**Diastolic BP**			
Less than 60 mmHg	65 (15.9)	12 (27.3)	0.057
More or equal 60 mmHg	343 (84.1)	32 (72.7)	
**Pulse rate**			
Less than 60/min	2 (0.5)	1 (2.3)	0.261
More or equal 60/min	404 (99.5)	42 (97.7)	
**Haemoglobin**			
Less than 12 g/dL	110 (18.8)	11 (20.8)	0.733
More or equal 12 g/dL	474 (81.2)	42 (79.2)	
**White blood cell**			
Less than 4.0x10^9^/L	382 (65.0)	30 (51.7)	0.045
More or equal 4.0x10^9^/L	206 (35.0)	28 (48.3)	
**Platelet**			
Less than 150x10^9^/L	494 (84.0)	49 (86.0)	0.700
More or equal 150x10^9^/L	94 (16.0)	8 (14.0)	
**Haematocrit**			
Less or equal 46%	552 (94.0)	49 (84.5)	0.012
More than 46%	35 (6.0)	9 (15.5)	
**AST** (3x upper normal 31U/L)			
Less or equal 93 U/L	36 (49.3)	9 (42.9)	0.602
More than 93 U/L	37 (50.7)	12 (57.1)	
**ALT** (3x upper normal 33 U/L)			
Less or equal 99 U/L	397 (77.5)	34 (65.4)	0.049
More than 99 U/L	115 (22.5)	18 (34.6)	
**Albumin**			
Less than 40 g/L	422 (81.9)	50 (94.3)	0.022
More or equal 40 g/L	93 (18.1)	3 (5.7)	
**Urea**			
Less or equal 6.7 mmol/L	504 (97.3)	45 (90.0)	0.019
More than 6.7 mmol/L	14 (2.7)	5 (10.0)	
**Creatinine**			
Less or equal 80 mmol/L	470 (90.9)	44 (88.0)	0.450
More than 80 mmol/L	47 (9.1)	6 (12.0)	

*Pearson chi square/Fisher exact test applied

Previous co-morbid, vomiting, diarrhea, pleural effusion, low systolic blood pressure, high hematocrit, low albumin and high urea were found as significant risk factors for severe dengue using simple logistic regression. However the significant risk factors for severe dengue with multiple logistic regressions were only vomiting, pleural effusion, and low systolic blood pressure. Table 3 showed the odds ratio using multiple logistic regressions.

**Table 3:** Significant factors associated with severe dengue (using multiple logistic regressions).

Variable	Adj OR	95% CI OR	χ^2^ statistic^a^ (df)^a^	P value^a^
Vomiting				
Yes	2.65	1.16,6.05	5.35 (1)	0.021
No	1.00			
Pleural effusion				
Yes	33.33	10.00,111.06	32.61 (1)	<0.001
No	1.00			
Systolic BP				
<90 mmHg	9.42	2.18,40.61	9.05 (1)	0.003
≥ 90 mmHg	1.00			

Adj OR = Adjusted odds ratio

^a^ Likelihood Ratio (LR) test

**Fig 2:**
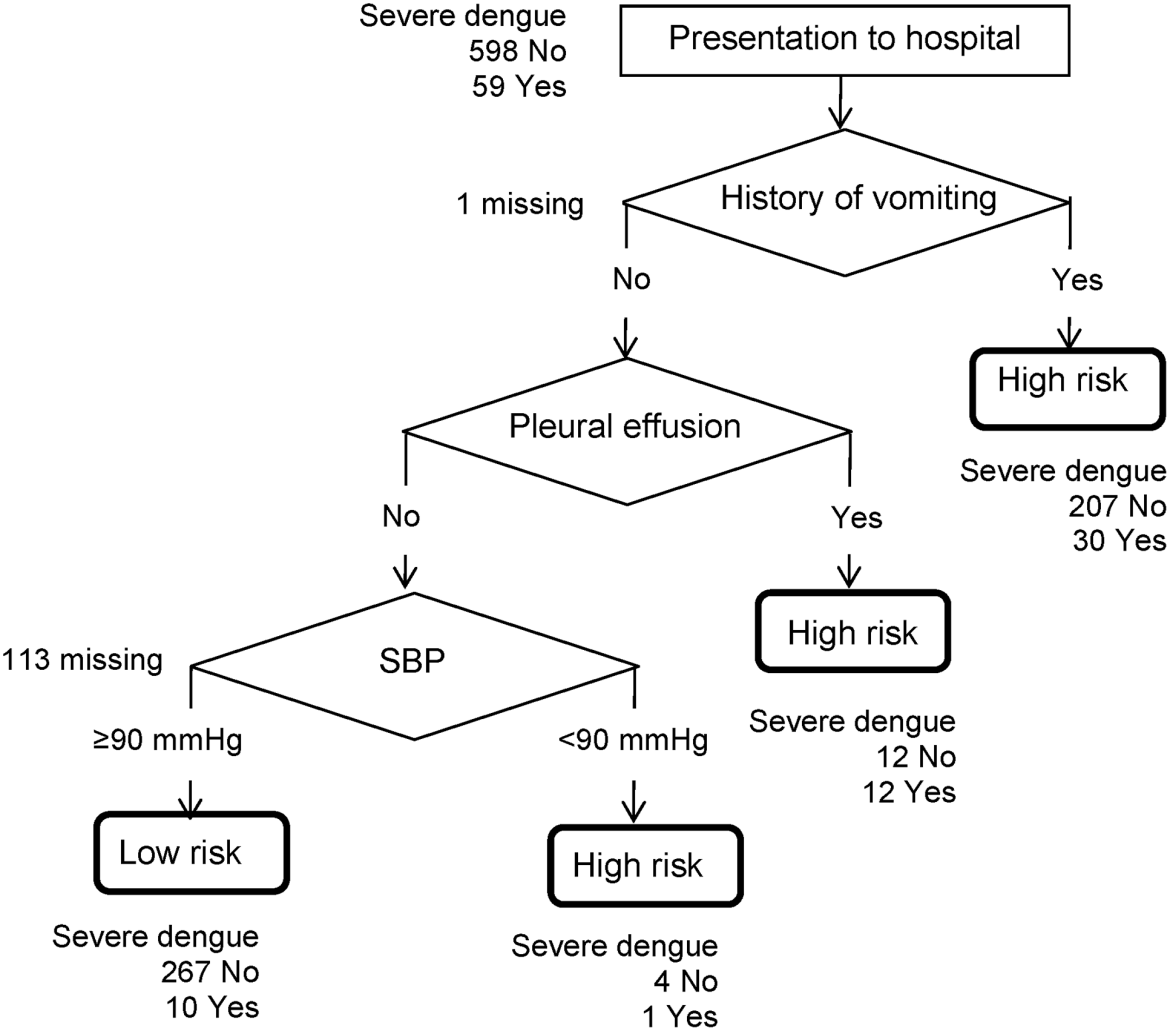
Decision tree for severe dengue infection.

Using those 3 risk factors, we plotted an algorithm for predicting severe dengue. Fig 2 showed the decision tree algorithm for severe dengue. When compared to the classification of severe dengue based on the WHO criteria using clinical diagnosis, the decision tree algorithm had a sensitivity of 0.81, specificity of 0.54, positive predictive value (PPV, the ability to predict dengue fever as severe dengue fever) of 0.16 and negative predictive value (NPV, the ability to predict dengue fever as non-severe dengue fever), of 0.96 as shown in Table 4.

**Table 4:** Comparison of decision tree with actual clinical diagnosis.

	Clinical diagnosis			
	Severe dengue			
	No	Yes	Total	
Decision tree				
Low risk	267	10	277	NPV = 0.96
High risk	223	43	266	PPV = 0.16
Total	490	53	543	
	Specificity = 0.54	Sensitivity = 0.81		

## Discussion

The WHO 2009 classification for Dengue Hemorrhagic fever and severe dengue has necessitated a large amount of hospital admission among those diagnosed with dengue fever [7, 8]. This has posed a huge impact on the country’s economic burden and has been responsible for large hospital bed occupancy, directly adding burden to the already saturated healthcare community[8]. The determining factors for progression into severe dengue or dengue hemorrhagic fever vary from studies[9, 10]. Though most of them correspond to the proposed warning signs in WHO guidelines, not all factors are needed to be present in predicting progression of disease into a more severe form[10]. The difference in these studies maybe attributed to the difference in cohort.

Kalayanarooj et al., noted that the overly sensitive nature of WHO 2009 classification would have created a 2 fold additional workload to medical personnel in his practice center in Bangkok Thailand [11]. This will cause more economical burden especially in endemic regions where resources are scarce[11, 12]. Another study by Yee-Sin Leo et al., Singapore suggested a 2–3 fold increase in workload if the hospital adopted warning sign -guided admission over the said hospital’s admission criteria and clinician’s judgement[13].

A lack of an evidence based diagnostic or decision algorithm for segregating clinically stable dengue patients from those who are at risk of developing severe dengue has been deterring effective patient management. Prognosticating dengue patients early upon presentation can help better channel healthcare resources and help in better clinical management of the patient[14].

Numerous other studies have been published on clinical and laboratory predictors for severe dengue. A study carried out in Pakistan, a country where Dengue is endemic, in the year 2011, with a sample of 640 patients to report the clinical parameters and pattern of Hemorrhagic complications, reported the presence of abdominal pain, purpuric rash, ascites, very low platelet count, significantly raised ALT and evidence of coagulopathy in the form of prolonged prothrombin time and activated partial thromboplastin time, have high predictability value for the development of DHF[15]. Comparison between DHF/DSS and DF cases revealed a significant difference in vomiting (p = 0.04), purpuric rash (p < 0.001), systolic blood pressure (p = 0.002), serum ALT (p < 0.001), hospital stay (p < 0.001), neurological involvement (p < 0.001) and coagulopathy (p < 0.001) between the two groups[15].

A publication by Lee et al in 2009 in Singapore found that history of mucosal bleeding, serum levels of urea and serum protein levels contributed to predicting early DHF. In the said study, a similar decision tree algorithm was postulated with a specificity of 46% and sensitivity of 100% using the factors mentioned to predict the severity of illness during presentation[13]. Yet another study done in Sri Lanka in the year 2006, to study the patterns of disease among adults hospitalized with dengue infections, where results from studies published from different countries such as, Singapore, Cuba, Thailand, Nicaragua, Bangladesh, and Taiwan was compared. A high proportion of patients with DHF had abdominal symptoms: diarrhea (32%), vomiting (68%) and abdominal pain (18.7%)[16]. The study concentrated on the outcome of dengue infection in adults and children. Overall, children had higher risk of developing shock, however, adults had higher risk of mortality[16]. A notable similarity in these studies are, determining factors in disease progression into severe dengue or DHF with significant predictive value are GI symptoms such as vomiting /diarrhea, deranged liver enzymes, serum protein levels and evidence of plasma leakage[13, 15, 16].

Plasma leakage and intrinsic coagulopathy are the pathological hall marks in dengue hemorrhagic fever (DHF)[17]. The key contributors implicated in pathogenesis of DHF are viral virulence, infection enhancing antibodies, cytokines and chemical mediators in the setting of intense immune activation though the exact nature of the event is yet to be fully understood [17]. Pleural effusions are the most common sign of plasma leakage in patients with DHF[18]. Pleural effusion was found in 62% of DHF cases one day after defervescence via ultrasonography, whereas thickening of the gallbladder wall and ascites were detected less frequently (43% and 52% of DHF cases respectively) and resolved more rapidly than pleural effusions in a study published by Anon Srikiatkhachorn et all[19]. Similarly, pleural effusion has also been quoted as a parameter to predict progression of disease in dengue fever[11, 17].

Other biochemical parameters have also been quoted to have predictive value on the severity of Dengue Fever. Soluble tumor necrosis factor receptor 80 had a sensitivity of 67% and NPV of 69% (green et all. 1999), free secreted NS I had a sensitivity of 72% and NPV69% (library et all 2002) platelet associated Gimp had a sensitivity of 49% and a specificity of 92%, and dengue viral load ad a NPV of 95% and NPV of 88%. However, these tests a not readily available at most health care center and some of them may take days to obtain results, hence these parameters are not practical for triage purposes[15].

In our study, we focused on narrowing down the admission criterions for predicting severe dengue at first presentation, hence reducing unnecessary admissions of stable dengue patients. The decision tree algorithm was designed after carefully studying data collected from a local public hospital. The advantage of our decision tree algorithm is the ease of use for clinicians in developing countries especially in district hospitals where expensive laboratory tests and equipment are often unavailable. Consistent with many other studies published as mentioned above, our results too suggests persistent vomiting at admission could be used as an indicator to predict development of severe dengue, similar to a finding of a previous multicenter study showing that persistent vomiting was one of the warning signs for severe dengue [7, 20]. Although the sensitivity of persistent vomiting for identifying severe dengue in adults was very low (6.0–23.0%), the specificity was as high as 93.0–96.0% and the negative predictive value was 82.0–97.0% [21].

There are several limitations to our study, one of which is that this is a single cohort study and the results may not be generalisable to others. Hence, a validation study of the proposed tool is needed in a different setting.

Our study only involved female patients. The data presented above were collected from a single ward from the study site, to ensure the quality of the data, as the patients recruited from the said ward was managed by the same consultant physician. Nevertheless, we were able to obtain a decent number of subjects for analysis. A decision tree algorithm study on dengue done in Singapore by VJ Lee et al, and published in September 2009 showed the sensitivity and specificity of 100% and 46% respectively. Based on the specificity, sample size calculation was done using single proportion sample size formula and the total sample size required was obtained by using the prevalence of disease. To achieve the precision of 0.05 for specificity of 0.46, we need at least a total sample size of 398 patients. For this study we retrospectively included 657 patients who were diagnosed with dengue who was admitted during the 4 months duration.

Though studies on difference in outcome of severe dengue among the male and female genders are scarce, a study by Katherine L. Ander et al did note a substantial and unexpected bias to males among dengue cases in Ho Chi Minh city, Vietnam[22]. However, the paper also cited healthcare seeking behavior may account for the gender bias as similar trend was not only observed in dengue cases but also the total admissions to the hospital which saw two times as many males as females was admitted in some years [18, 22]. Nyuen Tien Huy publishes a systematic review and meta- analysis on factors associated with dengue shock syndrome, quoted the association between being a female and risk of DSS is not fully understood however, may be explained by gender differences in health seeking behavior. Many other smaller hospital based studies have reported no difference in outcome among the dengue patient population [22].

## Conclusion

The decision tree algorithm based on the significant factors associated with severe dengue in this study showed high sensitivity and NPV in predicting patients with severe dengue that may warrant admission. This tool upon further validation study can be used to help clinicians decide on further managing a patient upon first encounter. It also will have a substantial impact on health resources as low risk patients can be managed as outpatients hence reserving the scarce hospital beds and medical resources for other patient in need. However, the patients who are not admitted and managed as outpatient will need continuous monitoring as outpatient as dengue is an evolving disease. The current practice in Malaysia, whereby once diagnosed with dengue fever and deemed stable, patients are discharged home equipped with dengue home care leaflet and dengue monitoring card. Patients are reviewed in the outpatient department on a daily basis until they are out of critical phase, before being discharged from follow up. Additionally, although the current study attempts to dissect patients with dengue into high and low risk, the act of admitting patients with WS according to the 2009 guideline itself, may have averted severe dengue. Information from this study, however, is important in allowing future researches to select group of patients that may benefit from step down management other than admission with intravenous fluid.
